# Investigating the effectiveness and cost-effectiveness of FITNET-NHS (Fatigue In Teenagers on the interNET in the NHS) compared to Activity Management to treat paediatric chronic fatigue syndrome (CFS)/myalgic encephalomyelitis (ME): protocol for a randomised controlled trial

**DOI:** 10.1186/s13063-018-2500-3

**Published:** 2018-02-22

**Authors:** Sarah Baos, Amberly Brigden, Emma Anderson, William Hollingworth, Simon Price, Nicola Mills, Lucy Beasant, Daisy Gaunt, Kirsty Garfield, Chris Metcalfe, Roxanne Parslow, Harriet Downing, David Kessler, John Macleod, Paul Stallard, Hans Knoop, Elise Van de Putte, Sanne Nijhof, Gijs Bleijenberg, Esther Crawley

**Affiliations:** 1 Centre for Child and Adolescent Health, Bristol Medical School: Population Health Sciences, University of Bristol, Oakfield House, Oakfield, Grove, Bristol, BS8 2BN UK; 2 Bristol Medical School: Population Health Sciences, University of Bristol, Canynge Hall, 39 Whatley Road, Bristol, BS8 2PS UK; 3 Computer Science, University of Bristol, Merchant Venturers Building, Woodland Road, Bristol, BS8 1UB UK; 4 Bristol Randomised Trials Collaboration, University of Bristol, Bristol, UK; 5 Bristol Medical School: Population Health Sciences, Oakfield House, Oakfield Grove, Bristol, BS8 2BN UK; 6 Department for Health, University of Bath, Bath, BA2 7AD UK; 7 Department for Medical Psychology, Academic Medical Centre (AMC) University of Amsterdam, Postbox 22660, 1100 DD Amsterdam, The Netherlands; 8 Department of Paediatrics, Wilhelmina Children’s Hospital, University Medical Centre, Utrecht, The Netherlands; 9 Radboud University Medical Center, Nijmegen, The Netherlands

**Keywords:** Paediatrics, Chronic fatigue syndrome, Myalgic encephalomyelitis, CFS/ME, CBT, E-health, Activity management, Online systems, E-therapy, E-counselling

## Abstract

**Background:**

Paediatric chronic fatigue syndrome or myalgic encephalomyelitis (CFS/ME) is a relatively common and disabling condition. The National Institute for Health and Clinical Excellence (NICE) recommends Cognitive Behavioural Therapy (CBT) as a treatment option for paediatric CFS/ME because there is good evidence that it is effective. Despite this, most young people in the UK are unable to access local specialist CBT for CFS/ME. A randomised controlled trial (RCT) showed FITNET was effective in the Netherlands but we do not know if it is effective in the National Health Service (NHS) or if it is cost-effective. This trial will investigate whether FITNET-NHS is clinically effective and cost-effective in the NHS.

**Methods:**

Seven hundred and thirty-four paediatric patients (aged 11–17 years) with CFS/ ME will be randomised (1:1) to receive either FITNET-NHS (online CBT) or Activity Management (delivered via video call). The internal pilot study will use integrated qualitative methods to examine the feasibility of recruitment and the acceptability of treatment. The full trial will assess whether FITNET-NHS is clinically effective and cost-effective. The primary outcome is disability at 6 months, measured using the SF-36-PFS (Physical Function Scale) questionnaire. Cost-effectiveness is measured via cost-utility analysis from an NHS perspective. Secondary subgroup analysis will investigate the effectiveness of FITNET-NHS in those with co-morbid mood disorders.

**Discussion:**

If FITNET-NHS is found to be feasible and acceptable (internal pilot) and effective and cost-effective (full trial), its provision by the NHS has the potential to deliver substantial health gains for the large number of young people suffering from CFS/ME but unable to access treatment because there is no local specialist service. This trial will provide further evidence evaluating the delivery of online CBT to young people with chronic conditions.

**Trial registration:**

ISRCTN registry, registration number: ISRCTN18020851. Registered on 4 August 2016.

**Electronic supplementary material:**

The online version of this article (10.1186/s13063-018-2500-3) contains supplementary material, which is available to authorized users.

## Background

Paediatric chronic fatigue syndrome/myalgic encephalitis (CFS/ME) is common in the UK, with estimated prevalence between 1 and 2.4% [1, 2]. CFS/ME is defined as disrupting and persistent generalised fatigue, diagnosed after routine investigations have failed to identify an alternative explanation for the fatigue [3, 4]. Young people with CFS/ME are disabled [5, 6] and 30% of them experience co-morbid anxiety and depression [7, 8]. They use significant healthcare resources [9] and put substantial burden on their families [10].

The National Institute for Health and Clinical Excellence (NICE) recommends that young people with CFS/ME are offered either Cognitive Behavioural Therapy (CBT, which focusses on strategies to identify, challenge and change fatigue-related cognitive processes and gradually resume activities), Graded Exercise Therapy (GET, which stabilises physical activity levels, before gradually increasing at a manageable rate) or Activity Management (a goal-oriented and person-centred approach which establishes a baseline for all activity, which is then increased) [4, 11]. CBT and GET are moderately effective in adults with CFS/ME [12–15]. There is good evidence from randomised controlled trials (RCTs) that CBT is effective for paediatric CFS/ME [16–19]. However, most young people in the UK do not have access to a local NHS specialist service offering CBT for CFS/ME. Some children in the UK are able to access treatment (GET or Activity Management) delivered as one face-to-face assessment with Skype follow-up appointments.

The FITNET (Fatigue In Teenagers on the interNET) trial carried out in the Netherlands [16] recruited 135 participants between 2008 and 2010, and showed that Internet-delivered CBT was effective compared to usual care at 6 months. Young people were more likely to have recovered; defined as no longer severely fatigued or physically impaired; attending school; and they perceived themselves as completely/nearly completely recovered (63% vs. 8%, relative risk 8.0, 95% CI 3.4–19.0; *p* < 0.0001). None of the published paediatric trials reported on cost-effectiveness and have either excluded young people with co-morbid mood problems [17] or have not been powered to investigate this group [16, 18, 19].

## Methods

The aims of this trial are to investigate whether Internet-delivered CBT, specifically designed for CFS/ME, (FITNET-NHS) is effective and cost-effective compared to a ‘usual care’ comparator of Activity Management (delivered via video call) for young people with CFS/ME who do not have access to a local specialist paediatric CFS/ME service. The trial is powered to explore effectiveness and cost-effectiveness in young people with mild to moderate co-morbid mood disorders.

This manuscript is in accordance with the Standard Protocol Items: Recommendations for Interventional Trials (SPIRIT) guidelines. See Additional file 1 for the Standard Protocol Items: Recommendations for Interventional Trials (SPIRIT) Checklist.

### Trial design

This is an RCT comparing FITNET-NHS with Activity Management for paediatric CFS/ME. An internal pilot study will be conducted with continuation of the trial based on achieving defined criteria. Integrated qualitative methods will be used to optimise recruitment and retention. Pilot data will be used in the effectiveness analyses unless changes to the interventions or trial design are significant.

### Setting and trial population

Young people will be assessed by their general practitioner (GP), referred for local paediatric assessment and investigated using NICE guidance [4]. If a diagnosis of CFS/ME is made and there is no local specialist paediatric CFS/ME service, GPs will be able to refer patients diagnosed with CFS/ME to the Bath specialist paediatric CFS/ME service. This is the standard referral pathway for out-of-area patients.

Young people will be eligible if they are: (1) aged 11–17 years, (2) diagnosed with CFS/ME (using NICE guidance [4]) and (3) do not have access to a local specialist paediatric CFS/ME service. Young people will be excluded if any of the following apply: (1) they are not disabled by fatigue (defined in eligibility screening), (2) their fatigue is due to another cause, (3) they are unable to complete video calls or FITNET-NHS online chapters or (4) they report pregnancy at assessment.

### Recruitment

The clinical team at the Bath specialist paediatric CFS/ME service will identify potentially eligible young people referred to the service and telephone them and their parents/carers to discuss treatment options, including the option to take part in the trial. Young people and parents/carers who are interested will be emailed an information pack including: an age-appropriate Patient Information Leaflet (PIL), and links to an online ‘consent to contact’ form and Revised Children’s Anxiety and Depression Scale (RCADS) questionnaire [20, 21]. The ‘consent to contact’ form and RCADS questionnaire will be completed using a secure electronic system used for data capture called Research Electronic Data Capture http://project-redcap.org (REDCap). After ‘consent to contact’ is completed an eligibility assessment will be carried out with both the young people and their parents/carers. Following this, the recruitment consultation is performed. The eligibility assessment and recruitment discussions usually take place during one telephone/video call; however, they can also take place over separate telephone/video calls if preferred. These discussions will be audio-recorded with consent/assent. The trial design and interventions will be explained; young people and their parents will be given the opportunity to read the age-appropriate PIL, ask questions and can have as long as required to make an informed decision. The researcher will seek age-appropriate consent/assent from the young person and parents/carers via the online REDCap system. Those who decide not to take part will be invited to talk to a qualitative researcher about their decision and verbal agreement will be recorded during the recruitment consultation. They will be offered a face-to-face assessment and treatment by the Bath specialist paediatric CFS/ME service and continue to receive standard medical care. Further consent for an interview will be obtained at the time of the interview.

### Randomisation

Participants will be randomised in a 1:1 ratio, by the research team, to receive either FITNET-NHS or Activity Management using an automated web randomisation service. Allocation will use minimisation to facilitate balance by age and gender, and preserve allocation concealment. Because of the nature of the intervention, it is not practical to blind either the participant, family or the clinical service to treatment allocation. GPs will be informed of the allocation.

### Interventions

**Fig. 1:**
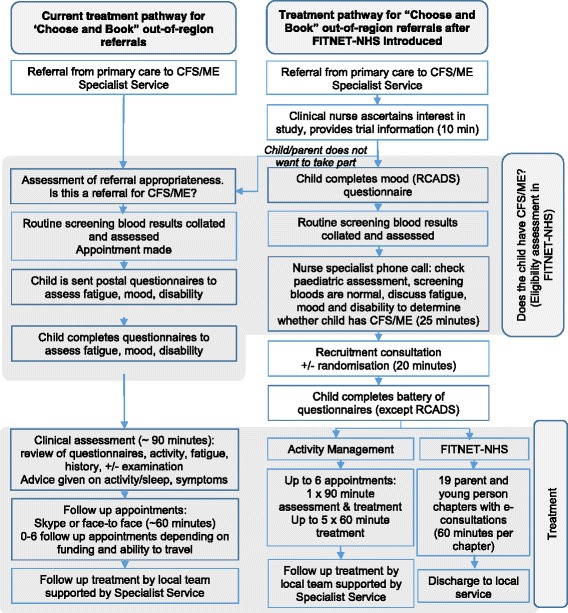
The trial treatment pathway identifies differences between the standard care (multidisciplinary treatment provided by the clinical care team as per NICE guidance) and research trial treatment pathways

The Bath specialist paediatric CFS/ME service will provide both treatment arms. Both interventions will be delivered so that participants receive treatment at home, online. The Activity Management arm is akin to usual care for out-of-area referrals, who would normally have an assessment (face-to-face) with follow-ups via video call. The difference with Activity Management delivered within the FITNET-NHS Trial is that participants do not need to travel for the initial assessment as this too is delivered via video call. See Fig. 1 for the standard referral and treatment pathway for out-of-area patients, compared with the trial processes.

#### Activity Management (comparator)

Activity Management via video call will be delivered by specialist therapists (occupational therapists, physiotherapists, psychologists, physicians and nurses). Participants will have three to six video appointments (one assessment and up to five follow-ups). Parent/carer attendance is optional.

During the assessment (around 90 min), the therapists: will discuss the different types of activity – including cognitive activity (high concentration and low concentration) – which vary according to age; carry out a detailed assessment of the individual’s current activity levels; and collaboratively agree a ‘baseline’, which is the average level of activity. Participants will receive information on CFS/ME, activity management, sleep and symptom management.

The first follow-up video call will be arranged 2 to 6 weeks after assessment depending on participant preference. If the patient still requires clinical care further follow-up video calls will be organised from 2 to 6 weeks later. During the follow-up video calls (around 60 min each) the therapist will review activity and sleep and help participants to problem-solve. Participants will be encouraged to increase activity between sessions.

Therapists will complete a checklist to indicate which mandatory, flexible and prohibited items were discussed at each session. These items are listed in Table 1.

**Table 1:** Outline of mandatory, flexible and prohibited elements from the Activity Management protocol

Mandatory	Discussing different types of activity (cognitive and physical) which vary according to age
	Finding a baseline level of activity (physical activities vary according to severity)
	Discussing different types of high-energy cognitive activities which require concentration, e.g. time at school, school work, reading, some crafts/hobbies, socialising and screen time (phone, TV, computer, other devices)
	Discussing physical activities, which vary according to severity (e.g. severely affected – sitting up in bed; mildly affected – running)
	Using paper/electronic diaries (including iPhone/iPad app ‘ActiveME’) to record time spent each day doing high-energy cognitive activities
	Increasing activity by 10–20% each week [4, 16]
	Problem solving
	Managing setbacks
Flexible	Advice on exercise
	Discussing a diagnosis and treatment of anxiety and/or depression
	Advice on medication (if required)
	Advice on symptom control (if required)
Prohibited	Detailed discussion of feelings, beliefs and how they change
	Diaries on feelings and their relationship with behaviour

Following the video calls the therapists will hand care over to the local nominated clinician in primary/secondary care. Therapists will discuss the case with the nominated clinician by telephone/letter (as normal clinical practice), ask for a review within 6–8 weeks and offer up to three telephone calls to advise on treatment options, overcoming barriers and symptom control. Local providers mostly offer face-to-face follow-up, some may use telephone.

#### FITNET-NHS (intervention)

FITNET is an Internet-delivered CBT package created for paediatric CFS/ME in the Netherlands [16, 17]. The programme has 19 psycho-educational and CBT chapters for young people and a parallel programme for their parents. Participants and their parents have separate accounts and log-ins. The psycho-educational chapters include information on: CFS/ME; the causes of CFS/ME; the relationship between CFS/ME, anxiety, depression and other illnesses; how diagnosis is confirmed; treatment for CFS/ME; how to explain CFS/ME to friends and what the future (without CFS/ME) is likely to look like.

The CBT section is activated by a clinical psychologist once the young person/parent has completed the psycho-educational chapters. Parental chapters explore and address parents’ beliefs and behaviours towards their child with CFS/ME, focussing on their role as carers. In participants younger than 15 years, parents/carers are supported to act as a coach. In those older than 15 years, parents/carers are encouraged to step back and support their child taking responsibility for their treatment. The CBT chapters focus on cognitive behavioural strategies with instructions on exercises for identifying, challenging and changing cognitive processes that contribute to CFS/ME and increasing self-efficacy with respect to fatigue, the ability to be active and work towards recovery. There are two protocols depending on the pattern of activity levels. If participants are identified as being relatively active with a varying level of activity, they first find their baseline before increasing slowly. If they are defined as being ‘low active’ with little variation in activity, they immediately start with increasing activity [17]. Chapters 1 to 4 introduce CBT and explain the role of therapists, present CFS/ME as a multifactorial model with predisposing, precipitating and maintaining factors and discuss the role of the family. Chapter 4 focus on treatment goals including the goal of full-time education and chapter 5 focusses on regulation of sleep-wake patterns. Chapters 6 to 19 focus on cognitive behavioural strategies with instructions on exercises on identifying, challenging and changing cognitive processes that contribute to CFS/ME. While participants are able to complete the chapters at their own pace, they are encouraged to work on, and complete, chapters before the next e-consultation.

The FITNET-NHS clinical psychologists will provide e-consultations (email exchange between therapist and participant on the FITNET platform) approximately every 1 to 2 weeks with timings negotiated between therapist and participant. In addition, participants and parents are required to complete homework/tasks (for example, sleep-wake, and thoughts and feelings diaries). These will be discussed in the e-consultations and used to support behaviour change. The therapist will work with parents and young people separately.

All FITNET-NHS clinical psychologists receive training in how to deliver CBT for CFS/ME and receive regular supervision from experienced CBT therapists.

#### Duration of treatment period

Those allocated to FITNET-NHS will receive treatment for approximately 6 months but this will vary depending on how long it takes participants and their parents to complete the online chapters.

Participants allocated to Activity Management will receive treatment for 3 to 6 months. It will vary depending on the number of sessions (three to six) in the treatment course and the gap between follow-up sessions.

After completion of the treatment, patients will be discharged from the Bath specialist paediatric CFS/ME service to their local GP.

#### Treatment adherence

For both trial arms we will use therapist-assessed treatment adherence with three possible ratings: (1) non-starter (no sessions attended), (2) started then stopped sessions, (3) majority (approximately 80% +) completer (all or majority of sessions attended in line with what is clinically relevant). New chapters within the FITNET-NHS programme are only unlocked by the therapist on completion of specific tasks/homework, and some chapters are opened only if clinically relevant. We will assume that participants and parents completing treatment find the interventions acceptable. Acceptability will also be assessed through qualitative interviews. Participants can withdraw from the trial at any time without giving a reason. The participant flow diagram is shown in Fig. 1.

### Outcome measures

The following data will be collected from participants at the clinical assessment: age, sex, post code, ethnicity, symptoms (Centers for Disease Control and Prevention (CDC) and NICE criteria), months of illness, and diagnoses of co-morbid illnesses. At baseline participants will complete the following questionnaires: SF-36 physical function subscale (SF-36-PFS) [22], fatigue (using the Chalder Fatigue Scale and the Checklist Individual Strength (CIS) [23] fatigue severity subscale), school attendance, RCADS [21, 24], pain Visual Analogue Scale [25], EQ-5D-Y (EuroQoL health-related quality of life questionnaire, Youth version) [26] and the Clinical Global Impression Scale questionnaire [27], the Cognitive Behavioural Responses to Symptoms Questionnaire (CBRSQ) [28, 29] and the Children’s Negative Cognitive Error Questionnaire Revised (CNCEQ-R) [30]. Parents will complete the following questionnaires at baseline: an adapted existing Healthcare Resource Use questionnaire to measure health service use and the adapted six-item Work Productivity and Activity Impairment Questionnaire General Health V2.0 (WPAI:GH) [31].

The primary outcome is disability measured using the SF-36-PFS 6 months after randomisation.

Secondary outcomes will be measured at 3-, 6- and 12-month follow-ups unless otherwise stated: SF-36-PFS measured at 3 and 12 months after randomisation; fatigue (Chalder Fatigue Scale and CIS); school attendance; RCADS); pain Visual Analogue Scale; Clinical Global Impression Scale; EQ-5D-Y; parental completed: Healthcare Resource Use questionnaire; WPAI:GH. All these measures are important and relevant domains [32] that are used in UK services, Child and Adolescent Mental Health Services (CAMHS) and/or tested in previous trials [16, 33, 34].

**Fig. 2:**
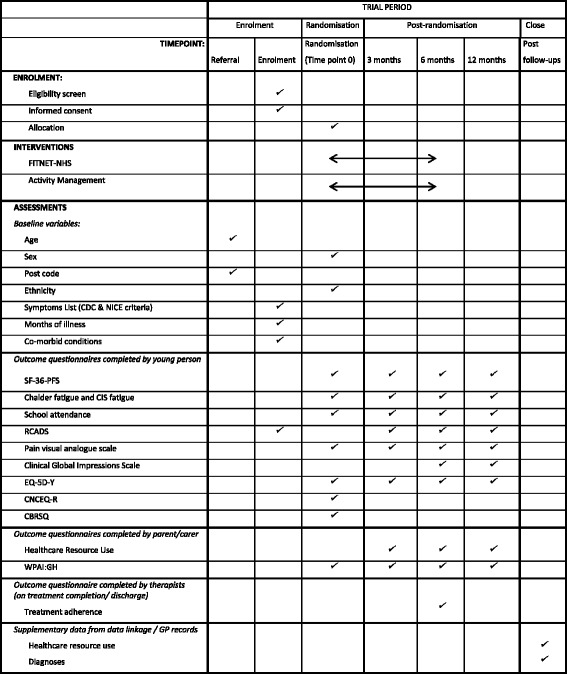
Standard Protocol Items: Recommendations for Interventional Trials (SPIRIT) Figure: schedule of enrolment, interventions and data collection [35]

All data will be collected on REDCap and anonymised at source. Table-based authentication will be used which utilises the storage of username/password pairs in a database table. Participants will be sent a web link to REDCap which will only allow access to their data. All data will either be stored on secure University of Bristol servers and or in secure locked cabinets. They will create a password which they will use each time they log in. The schedule of data collection is shown in Fig. 2, the SPIRIT Figure [35]. An email will be sent to participants with a link to complete questionnaires online. If these are not completed, two automated reminders will be sent and then we will telephone participants where possible. If this is not successful, an email with a link to a reduced set of questionnaires will be sent out (SF-36-PFS, Chalder Fatigue Scale, school attendance, EQ-5D-Y and Clinical Global Impression Scale).

### Sample size

Full trial: we plan to randomise 734 young people in the full trial. The study will proceed to full trial assuming the stop criteria (see below) are not met. Assuming 10% attrition (withdrawal or non-provision of primary outcome data) [16, 33], data on 660 young people will be available for the primary analysis. This gives 97% power at 1% significance to detect a 0.35-SD difference in treatment effect on the SF-36-PFS. The analysis of effectiveness in 198 young people with co-morbid mood disorders of anxiety and depression (30% of those recruited and with data available for analysis (after 10% attrition)) will have 80% power to detect a 0.4-SD difference at 5% significance. A difference of 10 points on the SF-36-PFS is considered to be the Minimal Clinically Important Difference (MCID) [36, 37]. The mean SF-36-PFS is 49.8 with SD 24.8 in young people with CFS/ME at assessment by the Bath specialist paediatric CFS/ME service (*n* = 1075). A 0.4-SD is 9.92 and, therefore, our trial is powered to detect the MCID of 10 in young people with co-morbid mood disorders of anxiety and depression.

We will perform sensitivity analyses on young people who fulfil the CDC diagnostic criteria. We estimate this will be 80% of those randomised (529 after 10% attrition) which will give us 98% power to detect a difference in treatment effect of 0.35 SD at 5% significance, which is at least the MCID.

### Outcomes and analyses of the internal pilot study

The internal pilot study will run for 12 months using integrated qualitative methods to determine feasibility of trial processes and acceptability of interventions. Defined criteria (see below) will be used to determine whether the study should proceed to full trial, in which case data from the internal pilot phase will be included in full trial analysis.

#### Feasibility of recruitment

We will present as a Consolidated Standards of Reporting Trial (CONSORT) flow chart the number (%) of those referred who: were contacted by the research team, were screened for eligibility, received recruitment consultation and consented. The number of referrals and the number recruited between different regions of the UK will be compared. Retention at 6 months will be calculated, defined as the proportion of participants providing outcome data between 5 and 9 months after randomisation.

Audio-recordings of eligibility and recruitment discussions will be analysed regularly, focussing on the interaction between recruiter and potential participant in terms of information provision, recruitment techniques, intervention preferences and trial participation decisions. This will be assessed in combination with data from in-depth interviews conducted with young people and their families soon after their consultation to explore provision and acceptability of patient information, reasons for accepting or declining participation, reasons for declining treatment allocation and dropouts, prior exposure to treatments, and beliefs, expectations and preferences about treatments. Screening logs will be scrutinised frequently to identify any recruitment issues, and interviews with members of the clinical and recruiting team will be undertaken to explore issues around trial feasibility if difficulties arise.

#### Acceptability of FITNET-NHS and Activity Management

Young people and their families who agreed to participate in the trial will be invited to an interview to assess their experience and acceptability of the trial interventions after having had exposure to them. Information will also be collected from interviews with trial staff on the feasibility and acceptability of delivering the interventions.

Sampling for interviews will ensure that a range of informants in terms of age, gender, ethnicity, geographical location, and severity of condition are included (maximum variation sampling), with further sampling being guided by emerging findings (theoretical sampling). Sample size will be determined by data saturation, i.e. where no new themes emerge. Topic guides developed from the academic literature and clinical experience will be used to ensure interviews cover the same issues while allowing new issues of importance to emerge. All interviews will be audio-recorded with consent using encryption software, transcribed verbatim and anonymised. Interviews will mostly take place via telephone or video call, or face-to-face where feasible.

Analysis of qualitative data will be an ongoing and iterative process commencing soon after data collection to inform further sampling and data collection. Transcripts will be imported into NVivo, systematically assigned codes and analysed thematically using techniques of constant comparison [38]. Individuals exhibiting contrasting attitudes (‘negative cases’) will be studied in detail. The perspectives of the individuals will be paramount, with careful account taken of the context within which the discussion takes place. To check coding reliability, a proportion of transcripts will be double coded and findings compared.

A purposeful selection of audio-recordings of eligibility and recruitment sessions will be analysed using content analytic methods [39] to describe in a structured manner what was said by whom and how often. More flexible Grounded Theory methods will be applied to identify common or divergent themes, particularly focussing on the impact of statements by the recruiter on patients. Targeted analyses focussing on certain sections of recruitment transcripts; for example, the interactions during which randomisation is offered, will be carried out to enable areas of difficulty to be explored in depth [40]. Sources of difficulties identified through the integrated qualitative research will be discussed with the trial management group and suggestions made to change aspects of the design, conduct, organisation or training of recruiters that could then lead to improvements in how the internal pilot study/full RCT is conducted.

#### Stop criteria

The stop criteria have been agreed with the Trial Steering Committee (TSC) prior to starting recruitment. The internal pilot study will not proceed to full trial if: (1) the recruitment rate is substantially below target during the last 6 months of the internal pilot study and if the qualitative data suggests that we cannot improve recruitment by changing recruitment methods or (2) the qualitative data suggests that the interventions are not acceptable to participants.

### Outcomes and analyses of the full trial

#### Effectiveness of FITNET-NHS compared to Activity Management

The primary outcome will be disability at 6 months measured by the SF-36-PFS.

We will compare the mean SF-36-PFS scores at 6 months according to randomised allocation among participants with measured outcomes, using multivariable linear regression adjusting for baseline values of the outcome, baseline age and gender. Similar regression analyses will be conducted for secondary outcomes (linear regression for numerical outcomes and logistic regression for binary outcomes). For the primary outcome, we will conduct sensitivity analyses in which we also adjust for any prognostic variables for which there is a baseline imbalance between intervention arms. Further sensitivity analyses will use imputation for missing data (if appropriate). Three-month and 12-month outcome data will be analysed similarly. We will present summary statistics for the SF-36-PFS (at baseline, 3 months, 6 months and 12 months) for the participants allocated to FITNET-NHS who did and who did not complete at least module 19, as an initial investigation of the benefit of the intervention among those participants who were able to engage fully with it. We will conduct sensitivity analyses estimating the effectiveness of FITNET-NHS compared with Activity Management for the primary outcome only restricted to participants who fulfilled the CDC diagnostic criteria for CMS/ME at the time of recruitment to the trial.

#### Analyses of effectiveness for those with co-morbid mood disorders

We will estimate the effectiveness of FITNET-NHS compared with Activity Management on the primary outcome in participant subgroups defined by the presence or absence of baseline anxiety or depression, by using the age- and gender-specific clinical thresholds for each subscale on the RCADS. Evidence that the intervention effect differs between subgroups will be examined by adding interaction terms to the multivariable linear regression model for the primary outcome only.

#### Cost-effectiveness of FITNET-NHS and Activity Management

Our primary economic evaluation will compare differences in NHS costs and health outcomes, measured in Quality-adjusted Life Years (QALYs), between participants randomised to FITNET NHS and Activity Management in a cost-utility analysis.

NHS costs will include the costs of delivering each intervention (including future licence fee costs). We will record the number of video consultations in the Activity Management arm and the number of e-consultations in the FITNET-NHS arm. At each follow-up time point (3, 6 and 12 months) parents will be asked to complete an adapted resource-use questionnaire to measure the young person’s health service use (e.g. GP, specialist care or medications) as well as educational service (e.g. school counsellor) and family expenses in the last 3 months. We will also quantify the impact of the young person’s health on parental work using the WPAI:GH. In addition, Hospital Episode Statistics (HES) data and the Mental Health and Learning Disabilities Data Set (MHLDDS) from the Health and Social Care Information Centre (HSCIC) will be used to measure paediatric and CAMHS use in both arms. Resource use will be valued using national unit costs where available.

We intend to use the EQ-5D-Y to calculate QALYs. A valuation tariff for the EQ-5D-Y is being developed but is not yet available [41]. Some researchers have used the adult valuation tariff to estimate an index score for the EQ-5D-Y, although this may misrepresent young people’s values [42]. At the time of analysis, we will use the EQ-5D-Y valuation tariff, if available.

Depending on the prevalence of missing cost and outcome data, we may use simple or multiple imputation methods in our primary analysis. We will use a threshold willingness-to-pay of £20,000 per QALY [43] to estimate the incremental net monetary benefits of FITNET-NHS. We will use non-parametric bootstrapping methods to calculate 95% confidence intervals and create a cost-effectiveness acceptability curve to estimate the probability that FITNET-NHS is cost-effective at varying willingness-to-pay thresholds. Between-group analyses of incremental costs, QALYs and net benefits will be adjusted for age, gender and baseline EQ-5D-Y score.

#### Analyses of cost-effectiveness for those with co-morbid mood disorders

In a secondary economic analysis, we will estimate cost-effectiveness acceptability curves for improvement in the primary outcome measure (SF-36-PFS). We will use net benefit regression to assess whether there is an interaction between cost-effectiveness and the presence of anxiety or depression at baseline. We will quantify non-NHS costs and compare them between trial arms to judge whether they strengthen or weaken the interpretation of the primary economic evaluation.

Missing data from parental healthcare resource-use questionnaires from general practice records will be collected through data linkage, and will be used to check accuracy of reported healthcare resource use and to determine how many young people develop other illnesses.

### Safety

We will prospectively collect data on serious and non-serious adverse events reported to the clinician or research team during the intervention and follow-up period. These will be reported according to the sponsor’s guidelines. We will investigate whether young people randomised to one arm are at higher risk of having a serious deterioration in health compared to another arm. We will define a serious deterioration in health as: (1) clinician-reported serious deterioration in health, (2) a decrease of ≥ 20 in SF-36-PFS between baseline and 3, 6 or 12 months or scores of ‘much’ or ‘very much’ worse on the Clinical Global Impression Scale or (3) withdrawal from treatment because of feeling worse.

Safety outcomes will be analysed by the Data and Safety Monitoring Committee (DSMC) at 11 months after the start of recruitment, before the trial progresses from internal pilot to full trial and when approximately 50% of participants have been recruited (estimated 23 months after the start of recruitment).

Following the baseline mood assessment (using RCADS), the research team will not routinely monitor depression or anxiety. Participants will be made aware of this and if they are worried about anxiety or depression, or disclose information about this during the course of the trial, they will be advised to discuss this with their routine care providers (e.g. GP, paediatrician), and clinicians will inform them about relevant services if appropriate. Participants will be made aware that if they report any serious problem outside of business hours, it may not be followed-up until business hours resume and they should contact their GP, paediatrician or emergency service.

## Discussion

Young people with CFS/ME should be offered referral to a specialist paediatric CFS/ME service [4]. However, most young people in the UK do not have access, or must travel very long distances to an NHS specialist service offering NICE-recommended treatments including Activity Management or CBT for CFS/ME. There is an evidence base for the using CBT to treat paediatric CFS/ME. However, none of the published paediatric trials have reported on cost-effectiveness. Although Internet-delivered CBT appears to be potentially useful, further evaluation is required before it is used within the NHS [44].

### Strengths and limitations

This will be the largest RCT conducted in paediatric CFS/ME. It is designed to investigate the effectiveness and cost-effectiveness of using FITNET-NHS, delivering online CBT, compared to Activity Management delivered via video calls, to treat paediatric CFS/ME in the UK. If effectiveness and cost-effectiveness are demonstrated for either arm, the NHS has the potential to deliver substantial health gains for the large number of young people suffering from CFS/ME but unable to access treatment because there is no local specialist service. If it is feasible this method could be used for other long-term conditions where young people do not have local specialist services. Results from the qualitative methods will tell us about patient experiences of the intervention content and mode of delivery, how to improve these types of interventions and how to deliver treatment from a single centre in the UK. Results will be disseminated as widely as possible including: open-access journals, conferences and public events. This is likely to benefit young people from other conditions, their families, clinicians and the NHS.

There is currently limited evidence of treatment effect in children with co-morbid mood disorders [45]. Most, but not all [46], studies in adults suggest that CBT is less effective in patients with co-morbid depression [47–49]. Anxiety and depression is common for young people with CFS/ME [8, 50, 51]. FITNET-NHS is designed to treat young people with co-morbid mood disorders as well as CFS/ME and this trial is powered to test whether the effects of FITNET-NHS differ in this subgroup of young people.

Our trial design includes usual care delivered by a specialist service. This is because it was felt to be unethical to offer no treatment to children in the control arm, though for many children in the UK ‘usual care’ is no treatment.

As Activity Management and CBT are behavioural interventions it is not feasible to blind participants or clinicians to allocation. However, the research team have worked to ensure the information sheets present the two treatments in a balanced way, and recruiters have had training to try and encourage participant equipoise. The analyses will be conducted by a researcher blinded to treatment allocation. As we are investigating CFS/ME, the outcomes are patient-reported outcomes. These outcomes are consistent with illness domains that are the most important to patients. The outcomes at follow-up are not reported to clinicians to reduce performance bias.

There is potential for contamination between interventions because of contact between therapists of two treatment arms (working in the same treatment centre); however, both treatment arms are protocolised and adherence will be checked.

If differences in effectiveness are found between the treatments, further research will be required to explore mechanisms of effectiveness.

### Trial status

Opened to recruitment November 2016. Recruitment is ongoing into 2020. For details see: www.bristol.ac.uk/fitnet-nhs/.

## Additional file

Additional file 1: SPIRIT: Standard Protocol Items: Recommendations for Interventional Trials guidelines. (PDF 129 kb)

## Abbreviations

CAMHS: Child and Adolescent Mental Health Services
CBRSQ: Cognitive Behavioural Responses to Symptoms Questionnaire
CBT: Cognitive Behavioural Therapy
CDC: Centers for Disease Control and Prevention
CFS: Chronic fatigue syndrome
CIS: Checklist Individual Strength
CNCEQ-R: Children’s Negative Cognitive Error Questionnaire Revised
DSMC: Data and Safety Monitoring Committee
EQ-5D-Y: EuroQoL health-related quality of life questionnaire, Youth version
FITNET: Fatigue In Teenagers on the internet
GET: Graded Exercise Therapy
GP: General practitioner
HES: Hospital Episode Statistics
HSCIC: Health and Social Care Information Centre
MCID: Minimal Clinically Important Difference
ME: Myalgic encephalomyelitis
MHLDDS: Mental Health and Learning Disabilities Data Set
NHS: National Health Service
NICE: National Institute for Health and Clinical Excellence
PIL: Patient Information Leaflet
QALYs: Quality-adjusted Life Years
RCADS: Revised Children’s Anxiety and Depression Scale
RCT: Randomised controlled trial
REDCap: Research Electronic Data Capture
SF-36-PFS: SF-36 physical function subscale
SPIRIT: Standard Protocol Items: Recommendations for Interventional Trials guidelines
TSC: Trial Steering Committee
WPAI:GH: Work Productivity and Activity Impairment Questionnaire General Health
